# Redundancy can hinder adult L2 grammar learning: evidence from case markers of varying salience levels

**DOI:** 10.3389/fpsyg.2024.1368080

**Published:** 2024-05-22

**Authors:** Panagiotis Kenanidis, Miquel Llompart, Sara Fernández Santos, Ewa Dąbrowska

**Affiliations:** ^1^Chair of Language and Cognition, Department of English and American Studies, Friedrich-Alexander-Universität Erlangen-Nürnberg, Erlangen, Germany; ^2^Department of Translation and Language Sciences, Universitat Pompeu Fabra, Barcelona, Spain; ^3^Department of English Language and Linguistics, University of Birmingham, Birmingham, United Kingdom

**Keywords:** redundancy, salience, artificial language learning, second language acquisition, grammar

## Abstract

Grammatical redundancy is a widespread feature across languages. Although redundant cues can be seen to increase the complexity and processing burden of structures, it has been suggested that they can assist language acquisition. Here, we explored if this learning benefit can be observed from the very initial stages of second language (L2) acquisition and whether the effect of redundancy is modulated by the perceptual salience of the redundant linguistic cues. Across two experiments, three groups of adult native speakers of English were incidentally exposed to three different artificial languages; one that had a fixed word order, Verb-Object-Subject, and two in which thematic role assignment was additionally determined by a low-salient (Experiment 1) or a high-salient (Experiment 2) redundant case marker. While all groups managed to learn the novel language, our results pointed towards a hindering role of redundancy, with participants in the non-redundant condition achieving greater learning outcomes compared to those in both redundant conditions. Results also revealed that this impeding effect of redundancy on L2 learners can be attenuated by the salience of the redundant cue (Experiment 2). In conjunction with earlier findings, the present results suggest that the effect of redundancy on L2 acquisition can be differentially manifested depending on the stage of L2 development, learners’ first language biases and age.

## 1 Introduction

A common feature among many natural languages is that they can express the same grammatical function multiple times within an utterance. Speakers’ preferences for encoding messages with more or less information have been linked to contextual predictability. Linguistic forms (e.g., function words, morphological markers) that are more probable in the context in which they occur carry less information (for listeners to infer the intended message) and, hence, are more redundant (Jaeger, 2010; Piantadosi et al., 2012; Fedzechkina et al., 2017; for a review see Jaeger and Buz, 2017). The tendency for probabilistic reduction of more contextually predictable linguistic forms has been documented across several domains, including phonology (Aylett and Turk, 2004, 2006), morphology (Kurumada and Jaeger, 2015; Fedzechkina et al., 2017) and referring expressions (Tily and Piantadosi, 2009), prompting a surge in research exploring its functional role in language processing and communication. Such research has provided accumulating evidence suggesting that redundancy can confer several advantages. Firstly, it is thought to facilitate communication in the presence of noise (Gibson et al., 2019) by enhancing the salience of the target linguistic information and making it more robust (Winter, 2014; Monaghan, 2017). Secondly, it enables the communication of less predictable or improbable sentence meanings, since the use of redundant linguistic materials can help reduce ambiguity and thus allow the comprehender to decode the intended message (Aylett and Turk, 2006; Jaeger, 2010). These two facilitatory effects come into play once the linguistic system has been acquired. However, redundancy is also assumed to benefit the learnability of the redundant cues (Leufkens, 2020), as well as of the entire linguistic structures in which they occur (Dittmar et al., 2008; Tal and Arnon, 2022). It is the latter of the three advantages of redundancy, that of learnability, the present study is concerned with, given that it holds important implications for both first language (L1) and L2 acquisition, as it predicts more accurate processing, earlier acquisition, and better learning rates despite the added complexity of redundancy. While previous research suggests that redundancy indeed plays a special role in L1 acquisition (Dittmar et al., 2008; Monaghan and Christiansen, 2008; Chan et al., 2009), results on L2 acquisition present a somewhat mixed picture.

Evidence for the facilitative effect of redundant linguistic cues for language learning comes from research on the processing of differential object marking (DOM) in Spanish, where direct objects that are both definite and human animates are marked with the marker *a*. Using a self-paced reading task, Jegerski (2015) found that near-native L2 Spanish learners displayed native-like sensitivity to the case marker only when the direct object was additionally marked with the preverbal clitic *lo* (e.g., *Verónica **lo** visita **al** presidente todos los meses* “veronica visits the president every month”; note that the acceptability of these sentences is subject to regional variation, as they are more common in Mexican than Peninsular Spanish). Additional converging findings are provided by studies employing artificial language learning experiments. For example, Taraban (2004) tested adults’ ability to induce gender-like categories either through distributional information (class 1 pseudo-nouns reliably co-occurred with the postpositions *eef*, *rog*, and *ast*, and Class 2 pseudo-nouns with the postpositions *foo*, *ilg*, and *tev*, irrespective of their suffixes, *-oik* or *-oo*) or when transparent morphophonological cues were also available (class 1 nouns ended in *-oik* and Class 2 nouns in *-oo*). After extensive training (2–4 days, for approximately 1 h each day) on short phrases aimed at familiarizing learners with the lexical items and the case markers, those who were exposed to the language containing reliable morphophonological cues outperformed participants who had to rely solely on distributional information, indicating that the presence of additional cues was strongly facilitative for the learning of gender categories. Similar findings have also been reported in studies with child participants (Brooks et al., 1993).

At the same time, however, L2 acquisition research suggests that grammatical cues that are redundant are likely harder to perceive and may even go unnoticed or be skipped over, particularly in the initial stages of the acquisition process (Schmidt, 2001; Ellis, 2006; VanPatten, 2006). This appears to be the case for both L2 comprehension and production. For instance, in a follow-up of her earlier study, Jegerski (2021) failed to find a positive effect of clitic doubling on the processing sensitivity to DOM in a group of intermediate Spanish L2 learners. In addition, strong evidence of learners’ limited processing or inattention to redundant cues comes from a series of studies on learned attention effects in L2 acquisition (Ellis and Sagarra, 2010, 2011; Sagarra and Ellis, 2013). In one of those studies (Ellis and Sagarra, 2010), L1 English beginning and intermediate L2 learners of Spanish were presented with sentences containing two markers of temporality, a lexical and a morphological one, that were either congruent or incongruent (e.g., *Ayer el profesor de violín practicó/practica el concierto en el conservatorio de música*, “Yesterday the violin professor practiced/practices the concert at the music conservatory”). Eye-tracking data showed that beginning L2 learners were insensitive to tense incongruencies and relied significantly more on adverbs while intermediate level learners attended more to verbs and showed greater sensitivity to incongruencies, yet still relied primarily on lexical cues. Regarding production, Fedzechkina et al. (2017) found that L1 English speakers who were exposed to a fixed word order miniature language with optional (in 67% of all sentences) case marking that was redundant tended to drop the case markers, thereby reducing the production effort. An overview of the experiments investigating the effect of redundant linguistic cues in L2 acquisition can be found in Appendix A.

Taken together, these results indicate that while redundancy may be advantageous once a linguistic system, or part of it, has been learned, this is not the case in initial stages of L2 acquisition (or in cases of increased complexity/difficulty) with redundant or less informative cues being omitted or not processed. Earlier research suggests that, when first exposed to a novel language (or under incidental conditions), the cues that L2 learners are more likely to focus upon are largely determined by their prior L1 experiences (McDonald, 1987; MacWhinney, 2001; Ellis, 2006). For example, studies examining thematic role assignment in transitive sentences have demonstrated that learners’ earlier experience with a morphologically impoverished L1 (e.g., English) leads them to over-rely on word order cues, hindering the processing and acquisition of morphosyntactic cues, such as case marking (Grey et al., 2014; Rogers et al., 2016; Kenanidis et al., 2023). Such processing strategies can have an even more detrimental effect on grammatical morphemes that are largely redundant given that such cues are generally not essential for the correct interpretation of sentences and, consequently, are often overlooked (VanPatten, 2006) or blocked by learners’ attention to cues that are more familiar to them (Wulff and Ellis, 2018).

Despite this, a recent study by Tal and Arnon (2022) showed that the presence of redundant case marking cues can facilitate learning of a novel grammatical structure, at least in children. This study involved one group of adults and one group of 7- to 9-year-old children, all native speakers of Hebrew. Half of the participants in each group were exposed to a semi-artificial language that had a fixed OSV word order, while the other half were presented with a language that had the same word order but in which objects were followed by a redundant marker (-*pazz*). The novel language consisted of six real Hebrew nouns, all of which featured the pseudo-suffix *-ig,* and two real verbs. Each sentence, therefore, consisted of three lexical items (e.g., *Rofeigpazz zayarig naga* “The painter points at the doctor”). All learners were initially exposed to the noun labels and to a small number of sentences, allowing them to familiarize themselves with the novel language, and were subsequently tested on their ability to comprehend, via a picture selection task, and produce, by means of a picture description task, the OSV constructions. Despite having to process and learn an additional cue, child learners of the redundant language displayed better performance in comprehension (and, though not significantly, in production) of the novel structure, while this was not the case for the adult group. This differential effect of redundancy can be seen to constitute evidence in line with the Linguistic Niche Hypothesis (Lupyan and Dale, 2010), according to which redundancy can facilitate language acquisition in children by offering them multiple linguistic cues to meaning and minimizing their reliance on extralinguistic/pragmatic cues, but not in adult L2 learners, whose pragmatic knowledge about the world can allow them to reconstruct underspecified meanings, making the presence of redundant cues unnecessary and dispreferred.

Nevertheless, the findings in Tal and Arnon (2022) should be interpreted with caution, as is in fact acknowledged by the authors. This is mainly because their results for the adult learners are not conclusive. While a significant effect of condition and no interaction between condition and group emerged in the omnibus regression model on the comprehension data, indicating that the positive effect of redundancy might have been present in both groups, follow-up by-group analyses showed that the beneficial effect of redundancy was significant only for children (65% vs. 91% correct). Adult performances in the two conditions were numerically closer to each other and also closer to ceiling. Production results showed an overall similar pattern (children: 69% vs. 82%; adults: 85% vs. 95%), although no significant effects emerged in the regression model.

One possible reason behind Tal and Arnon’s (2022) difficulty in obtaining clearer results for novice adult learners might have to do with the use of a semi-artificial linguistic system in which lexical items were already familiar to learners, leading this population to near-ceiling effects. It is also conceivable that such a system actually tapped into more advanced, rather than early, L2 stages or even into language processing facilitation instead of actual learning. In this study, we attempt to extend earlier findings on the role of redundancy in L2 acquisition by testing its effect during the very earliest stages of L2 acquisition and for adult native speakers of a morphologically poor language, namely English.

### 1.1 Salience in L2 acquisition

Another factor that is considered to play a major role in the processing and learning of novel linguistic cues during the earliest stages of L2 is their perceptual salience (Goldschneider and DeKeyser, 2001; Schmidt, 2001; Ellis, 2006, 2017). This refers to certain properties that make such cues stand out from their surrounding items, capturing learners’ attention and becoming more noticeable (Rácz, 2012, 2013). Hence, the greater the salience of the cue or item is, the more sensitive learners are to it. Conversely, low perceptually salient cues, such as inflectional morphology and function words, tend to be more difficult to perceive and to learn. This has been identified as one of the main reasons why L1 speakers of English and Chinese tend to show greater sensitivity to temporal adverbs over verbal inflections when presented with L2 expressions or sentences containing both cues, with the low-salient inflectional morphemes being overshadowed and less readily learned (Ellis and Sagarra, 2010, 2011). Yet, naturally, not all grammatical morphemes exhibit the same level of salience and, thus, are not equally susceptible to learnability issues. This is demonstrated by a study by Simoens et al. (2017), where it was found that adult learners are more likely to attend to and process nonce possessive suffixes that appear in conjunction with possessive pronouns when these suffixes were highly salient (*his hotel-olp*) than when their salience was lower (*her hotel-u*). It is worth noting in this connection that the redundant grammatical marker used in Tal and Arnon (2022), *pazz*, was highly salient, as it consisted of three phonemes forming a heavy syllable (CVC). In addition, its salience was further enhanced by contextual information. Specifically, participants were exposed to sentences of a semi-artificial language with real Hebrew verbs and nouns (all nouns had the nonce suffix *-ig*, irrespective of whether they functioned as subjects or objects) in which *pazz* was the only fully novel lexical item. Therefore, it is possible that the effect of redundancy observed in Tal and Arnon (2022) may have been amplified by the high salience of the redundant case marker. A relevant question is thus whether the same effect will still arise if less salient grammatical morphemes are used.

### 1.2 The present study

In the current study, we report on two experiments that aim at investigating whether the presence of redundant case marking cues facilitates learning of novel grammatical structures, as well as how the level of perceptual salience of these cues might influence the potential impact of redundancy on learning. To do that, we capitalize on artificial language learning paradigms. Crucially, a fully artificial language, rather than a semi-artificial one, was employed in order to limit the influence of prior L1 knowledge on grammar learning and to reduce the possibility of further increasing the salience of the novel grammatical markers due to them being the sole unfamiliar features (Godfroid, 2016; Leow, 2018). In Experiment 1, two groups of adult English speakers were exposed to two different artificial languages that had the same fixed word order (VOS), but differed only in that in one of them grammatical role assignment was marked by redundant case marking cues, which appeared in the form of relatively low-salient suffixes on both subjects and objects. In Experiment 2, a new group of learners was exposed to sentences of yet a different artificial language, which as in Tal and Arnon (2022), had a highly salient redundant case marker (*pazz*) only for objects. Finally, by virtue of using a new artificial language learning paradigm, and in order to replicate the results of previous studies using artificial linguistic systems, we re-examined whether adult native speakers of English can attain advanced proficiency levels in a language with a novel word order (VOS) under incidental exposure conditions.

Given the limited complexity of the language’s grammatical structure, with redundant case marking and the lack of constituent order flexibility rendering word order a fully sufficient and reliable cue to grammatical role assignment, it was expected that participants in both groups would achieve high levels of grammar learning. Our predictions regarding the effect of redundancy on learning were less clear-cut. On the one hand, if redundant cues do indeed increase learnability, this should be reflected in superior performance in the redundant case marking groups in both experiments relative to the no case marking group. On the other hand, if redundant grammatical cues are processed only after the nonredundant ones have been processed (VanPatten, 2006), and given L2 learners’ difficulty with processing and learning novel case markers (Rogers et al., 2016; Kenanidis et al., 2023), then we would expect no differences between groups. Finally, if the salience of the redundant case marking cues influences their detectability by learners, then we would expect to find stronger learning in the redundant case marking group of Experiment 2 compared to the low salience redundant group of Experiment 1.

## 2 Experiment 1

### 2.1 Method

#### 2.1.1 Participants

A total of 58 adult participants (*M*_age_ = 29.45, SD_age_ = 8.15; female = 32) were invited via Prolific, an online recruitment platform, to complete the study in exchange for monetary reimbursement. Thirty of them were randomly assigned to the redundant case marking group (*M*_age_ = 29.73, SD_age_ = 7.96; female = 17) and the remaining 28 to the no case marking group (*M*_age_ = 29.14, SD_age_ = 8.48; female = 15). Prolific’s screening criteria were used to recruit participants who were monolingual native speakers of English and resident in the UK at the time of testing. The study was carried out in accordance with the Declaration of Helsinki. All participants were asked to provide informed consent via an online form before the test session and were compensated upon completing the experiment.

#### 2.1.2 Materials and procedure

##### 2.1.2.1 Artificial language

Participants were auditorily exposed to Kesadalo, an artificial language that was modeled after Kepidalo (Kenanidis et al., 2023). The artificial language consisted of ten nonce words, six nouns (*alg*, *velg*, *ird*, *prad*, *olb*, *flub*) that denoted aliens and four verbs (*mulek*, *dolek*, var*ek*, *birek*) which referred to four different transitive actions (catapult, chase, jump over, approach). For participants in the redundant case marking condition, both nouns were always marked for case, with the subject of the sentence taking the suffix *-i*, and the object carrying the suffix *-o*. All sentences had a Verb-Object-Subject word order, a syntactic pattern that is rarely attested cross-linguistically (in 3% of the world’s languages; Tomlin, 1986) and does not occur in English. Example sentences for the no case marking and the redundant case marking conditions are illustrated in (**1a**) and (**1b**), respectively.

For each of the two experimental conditions, a set of 120 novel sentences was generated using the Google Cloud Text-to-Speech service. Short animated scenes, each depicting the meaning of a different sentence, were constructed to accompany the auditory stimuli. The experimental stimuli and tasks created and used in this study are available on Gorilla Open Materials.^1^

**(1) a.** *Varek velg alg*

**b.** *Varek velg-o alg-i*

jump-over velg-ACC alg-NOM

‘The alg is jumping over the velg.’

**Figure 1:**
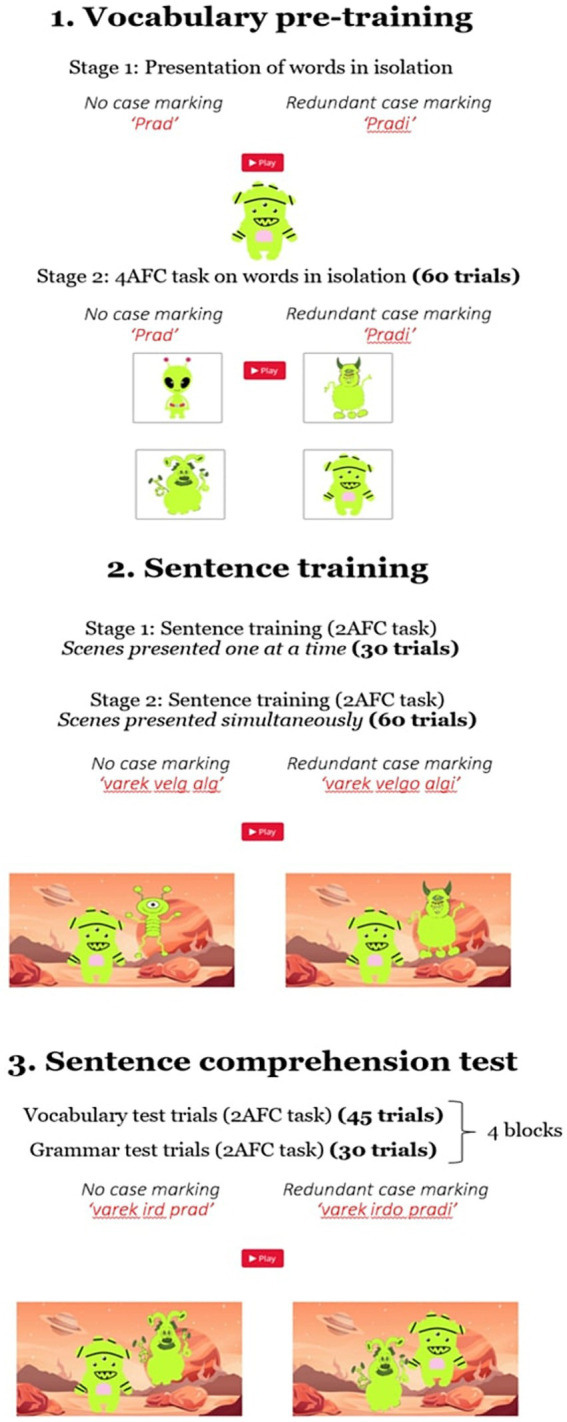
Summary of the experimental design. Pictures are still images of the visual stimuli used in the experiment.

The study was conducted online via the Gorilla experimental builder (gorilla.sc; Anwyl-Irvine et al., 2020) and was divided into three phases: (i) vocabulary pre-training, (ii) sentence training, and (iii) sentence comprehension test. A summary of the study design is provided in Figure 1.

##### 2.1.2.2 Vocabulary pre-training

Upon providing their informed consent, participants in both groups were introduced to the lexical items of the artificial language (stage 1). First, the ten nonce words were auditorily presented in isolation, one at a time, while their corresponding alien (for nouns) or action (for verbs; performed by two grey shapes, a square and a circle) was displayed on the screen. Each nonce word was presented only once.

Following this stage, additional vocabulary training was provided, this time in the form of a 4 alternative-forced-choice (4AFC) task (stage 2). During this stage, four images were presented, one in each screen quadrant, while a nonce word referring to one of them was played. Participants could initiate each trial by clicking on a ‘Play’ button that appeared on the top of the screen. Upon hearing the audio stimulus, they were instructed to choose the image they thought corresponded to the word they heard and were provided with feedback on their responses, followed by a presentation of the target image and nonce word.

Each lexical item served as a target six times, yielding a total of 60 trials. When the target item was a verb, participants were shown four short dynamic scenes in which the actions described by the four novel verbs (one target and three distractors) were depicted being performed by two geometric shapes, whereas in trials in which a noun was the target word, static images of four different aliens were displayed on the screen. For the redundant case marking condition, in both stages of the Vocabulary pre-training, all nouns were presented in their nominative form (e.g., *alg-i*). Participants were allowed to move on to the next phase of the study, regardless of their performance on this task.

##### 2.1.2.3 Sentence training

Following pre-training, participants in both conditions were exposed to the novel artificial language by means of a two-alternative forced-choice task (2AFC), which was also split into two separate stages that differed in their level of difficulty. The first stage consisted of 30 trials, each of which involved the presentation of two sentences, a target and a distractor, and their corresponding visual scenes. In each trial, one of the two scenes appeared on one side of the screen (for 4,500 ms), while the sentence describing it was played. After it disappeared, a new scene was displayed on the other side of the screen (also for 4,500 ms) and the second sentence was presented. Finally, both scenes re-appeared simultaneously, on the side of the screen where they previously appeared on, and a sentence matching only one of the depicted scenes was played. Participants were allowed to replay this final sentence once if they wanted. Crucially, the two scenes constituted minimal pairs as they differed only in a single aspect (Figure 1, 2. Sentence training); either in one of the two aliens (noun trials) or the action performed by the two aliens (verb trials). The decision to present the two visual stimuli one at a time was intended to make the contrast between them more explicit, accelerating the vocabulary learning process. Each of the ten novel lexical items served as the critical aspect in which the two scenes differed three times, amounting to 18 noun and 12 verb training trials. Participants were instructed to select the scene that the sentence referred to and received feedback on their responses. The side of the first appearance and the side of the matching scene were counterbalanced.

The second stage of the sentence training phase involved the presentation of 60 additional artificial language sentences and was identical to the final part of the first stage, with the two scenes immediately presented side-by-side on the screen. The two scenes were played in a loop giving participants ample time to process the information input. In this stage, participants completed 27 noun trials and 33 verb trials. Hence, across both stages of the sentence training phase, a total of 90 trials were administered, half of which tested noun learning and half verb learning. The sentences and scenes were the same for both experimental groups, except that for the redundant case marking group all nouns were case marked.

##### 2.1.2.4 Sentence comprehension test

Upon completing the sentence training phase, participants proceeded to the test phase of the study. In this phase, they were presented with yet another 2AFC task which was similar to the last training phase but in which, critically, no feedback on accuracy was given. The sentence comprehension test comprised 150 sentences. Of these, 90 were the same as the ones participants heard in the sentence training phase and were used for testing vocabulary learning. As in the previous phase, half of the sentences were used for testing noun learning and half for testing verb learning. The remaining 60 sentences aimed at assessing participants’ grammatical comprehension and, therefore, constitute the main focus of the present study. In all grammar test trials, two scenes were presented to participants in which the agent/patient roles of the nouns were reversed.

All 150 of the test sentences were repeated twice, for a total of 300 sentences. The test phase was divided into four blocks of 75 trials, each with 45 vocabulary and 30 grammar test trials. In each trial, participants heard a sentence and were instructed to choose the video that corresponded to it. Although no immediate feedback on response accuracy was given, to keep participants motivated, a total score showing the number of correct responses was presented at the end of each block, along with a message prompting them to beat their score in the next block.

### 2.2 Results

All analyses reported in the current study were conducted in R (version 4.3.1; R Core Team, 2022). For each of the artificial language tasks, participants’ responses were scored as correct (1) or incorrect (0) and, following that, individual scores were computed as the percentage of correct responses. For the sentence comprehension test, two separate scores were calculated for each participant, one for performance on the grammar test trials and another for performance on the vocabulary test trials.

The performance of the two groups in the training phases of the study is summarized in Table 1. The difference in performance between the two groups was found to be significant only in the first stage of the sentence training phase, according to a Mann–Whitney U test (*U* = 272, *p* = 0.02; Vocabulary pre-training: *U* = 408, *p* = 0.86; Sentence training Stage 2: *U* = 346, *p* = 0.25; Sentence training Stages 1 & 2: *U* = 316, *p* = 0.11). The lack of difference in the pre-training phase indicates that, before the presentation of sentences, participants in both groups had almost equal knowledge of the individual lexical items.

**Table 1:** Mean performance (%) in the training phases in the two conditions.

	Redundant case marking group					No case marking group				
	Mean	SD	Median	IQR	Min-Max	Mean	SD	Median	IQR	Min-Max
Vocabulary pre-training stage 2	53.7	19.2	56.7	22.5	16.7–90	55.8	17.6	55	17.5	21.7–95
Sentence training stage 1	76.9	15.1	83.3	22.5	33.3–96.7	84.4	12.9	90	13.3	56.7–96.7
Sentence training stage 2	70.4	15.8	65	25.8	41.7–98.3	75.8	19	82.5	35.4	41.7–100
Sentence training stages 1 & 2	72.6	14.2	71.1	20.6	46.7–97.8	78.7	15.4	83.3	26.7	52.2–97.8

**Figure 2:**
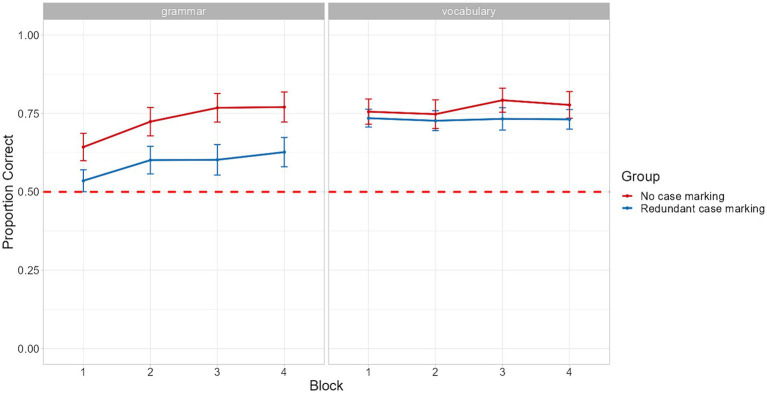
Performance across the four blocks of the sentence comprehension test. The red dashed line represents chance performance and error bars, calculated over by-subject means, represent standard errors of the mean.

Performance on the sentence comprehension test is shown in Figure 2. To probe the relationship between accuracy on vocabulary (sentences differing in one element, as in Figure 1, 2. Sentence training) and grammar test trials (Figure 1, 3. Sentence comprehension test), Spearman correlations were computed across all participants as well as separately for each group. A robust correlation was observed overall (*rho* = 0.76, *p* < 0.01), yet the two scores appeared to correlate more strongly in the no case marking group (*rho* = 0.85, *p* < 0.01) than for the redundant case marking group (*rho* = 0.58, *p* < 0.01).

To evaluate whether participants in both groups were able to learn the grammatical structure of the novel artificial language and whether the presence of redundant case marking had a facilitative or a hindering effect on their learning outcomes, we fitted a mixed-effects logistic regression model (Jaeger, 2008), which accounts for the binary nature of the dependent variable, to the grammar data from the sentence comprehension test. In order to control for differences in vocabulary knowledge among learners in both groups, a composite vocabulary learning score, consisting of the sum of scores on both stages of the Sentence Training phase, was also entered as a fixed effect. The model was implemented using the lme4 package (version 1.1–30; Bates et al., 2015) in R. Response accuracy was modeled as the categorical (correct = 1, incorrect = 0) dependent variable, and Group (effect coded as redundant case marking group = −0.5 and no case marking group = 0.5), Block (effect coded as block 1 = −1, block 2 = −0.5, block 3 = 0.5 and block 4 = 1), Vocabulary Learning (centered and scaled) and all possible (two- and three-way) interactions between them were entered as fixed effects. Centering the factor variables with this coding scheme allows for the intercept to be interpreted as the overall average of both groups across blocks (i.e., grand mean) and the fixed effects (and their interactions) as main effects, as in an ANOVA. The model included the maximal converging random effects structure justified by the design (Barr et al., 2013; Bates et al., 2015). Post-hoc analyses with Bonferroni corrections were conducted using the emmeans package (version 1.8.1–1; Lenth, 2022). Odds ratios and 95% confidence intervals for the fixed effects of the model were estimated using the tab_model function from the sjPlot package (version 2.8.11; Lüdecke, 2018). Variance inflation factor (VIF) scores calculated using the car package in R (version 3.1–2; Fox and Weisberg, 2019) did not indicate any multicollinearity problems among the predictor variables (VIFs <2.00). The results of all post-hoc analyses are shown in Appendix B. All data and R scripts for analyses can be found at: https://osf.io/v82j4/?view_only=14f5f4d603a84cecb1dbf183d6d73e8a.

Inspection of the model (Table 2) revealed a significant positive intercept, suggesting that in both conditions, learners chose the correct scene more often than would be expected by chance, providing evidence of learning of the target grammatical structure. Furthermore, the model found main effects of both Vocabulary Learning and Block indicating that response accuracy increased across blocks and that participants with higher vocabulary learning scores in the sentence training phase exhibited better grammar learning outcomes. A significant interaction between the two variables also emerged, for which a post-hoc analysis showed that the effect of Block was stronger for participants who performed better on the earlier lexical training trials but was not significant for those who achieved low scores (−1SD).

**Table 2:** Results of the mixed-effects model for response accuracy in the grammar trials of the sentence comprehension test.

Variable	$\hat{\beta }$	SE	*z*	*p*	Odds ratios (CI)
(Intercept)	1.18	0.17	6.77	<0.001	3.25 (2.31–4.57)
Block	0.65	0.10	6.39	<0.001	1.91 (1.57–2.33)
Group	0.74	0.34	2.17	0.030	2.09 (1.07–4.07)
Vocabulary learning	1.12	0.17	6.45	<0.001	3.07 (2.18–4.32)
Group: block	0.40	0.19	2.09	0.037	1.49 (1.02–2.17)
Group: vocabulary learning	1.09	0.34	3.20	0.001	2.99 (1.53–5.85)
Block: vocabulary learning	0.54	0.10	5.24	<0.001	1.71 (1.40–2.09)
Group: block: vocabulary learning	0.50	0.20	2.54	0.011	1.65 (1.12–2.42)
*Random effects*	*Variance*	*SD*			
Item	0.06	0.25			
Participant	1.45	1.21			
Group | item	0.08	0.28			
Vocabulary learning | item	0.02	0.15			
Block | participant	0.34	0.59			

**Figure 3:**
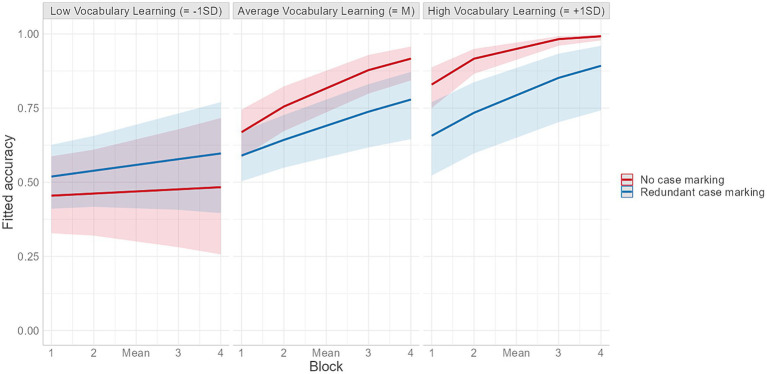
Fitted accuracy as a function of vocabulary learning and block for the two learning groups.

Most importantly for our central research question, the results of the model yielded a main effect of Group, which, as indicated by the positive regression coefficients, suggests that accuracy was higher for the no case marking group than the redundant case marking group. In addition, significant interactions between Block and Group and Group and Vocabulary Learning were also observed. Regarding the former interaction, post-hoc analyses showed that, overall, the effect of Group became more pronounced over time and the difference in performance between the two groups was found to be significant in blocks 3 and 4. Post-hoc analyses of the interaction between Group and Vocabulary Learning revealed that the difference in grammar learning outcomes across the two conditions was larger for participants who scored higher for vocabulary in the earlier training phase and was not significant for participants who exhibited low vocabulary learning scores (−1SD). These findings were confirmed by a significant three-way interaction between these variables (Figure 3). Subsequent post-hoc pairwise comparisons showed that the differences in performance across conditions emerged only for learners who achieved average (M) and high vocabulary scores (+1SD), and that for this latter subgroup of participants the difference was significant throughout all four blocks (see Appendix B).

### 2.3 Discussion

The results of Experiment 1 showed that learners in both groups exhibited strong learning effects of the artificial language’s grammatical structure, thereby providing corroborating evidence for learning of novel word order patterns under incidental exposure conditions (Williams and Kuribara, 2008; Grey et al., 2014). However, performance was still far from ceiling, even in block 4 of the experiment (62.7% for the redundant case marking and 77% for the no case marking group). Importantly, these learning effects were not observed to the same extent for all participants but were instead found to be modulated by their level of knowledge of the artificial language’s lexicon prior to the comprehension test. Furthermore, the strong relationship between grammatical and lexical knowledge appeared to hold for both groups, with participants with low vocabulary learning scores showing only limited grammar learning and, on the contrary, those who had better vocabulary knowledge performing near ceiling in the final block of the experiment (Figure 3).

Before discussing the main findings about grammar learning, there is one additional issue related to vocabulary learning that warrants mention. Specifically, while the two groups performed equally well in the pre-training and the second stage of the sentence training phase, their performance was found to diverge in the first stage of that phase. However, the difference in performance between groups was found to dissipate already from the second stage of the sentence training phase. Furthermore, in the first two blocks of the Sentence Comprehension test, the two groups achieved almost equal levels of accuracy in the vocabulary test trials (Figure 2), indicating that any differences in grammar learning during the main task are unlikely to have been caused by differences in knowledge of vocabulary items.

Regarding the effect of redundancy on novel grammar learning, the analyses presented here suggest that at the early stages of exposure, redundant case marking tends to be more hindering than beneficial. This effect appears to become stronger after only minimal exposure to the language and for learners who had previously demonstrated better knowledge of the language’s vocabulary. Hence, overall, the results of Experiment 1 point rather conclusively towards an impeding effect of redundant case marking on early L2 learning.

It is important to note that this pattern of performance appears to run counter to the general pattern of results observed in Tal and Arnon (2022) to a certain extent. In that study, the presence of redundant grammatical cues did not negatively interfere with the learning of a new language with a word order pattern that was different from the learners’ L1’s canonical order. In fact, performance by the adult group in the redundant condition was numerically higher in both comprehension and production. Several explanations can be invoked to account for this discrepancy. First, the patterns of performance could be partly attributed to participants’ L1 background. Specifically, while learners in the present study were native speakers of English, a strict word order language, Tal and Arnon’s participants (2022) were L1 speakers of Hebrew, a language which is morphologically richer than English and which, although it does not mark the objects with a suffix, does have overt case marking.^2^ A second potential reason pertains to the nature of the artificial languages used in the two studies, with participants in the present study being exposed to a fully artificial language as opposed to the semi-artificial language employed in the previous study. As a result, on top of learning the grammatical structure, here, participants were also tasked with learning the vocabulary of the language. Finally, and most importantly, the diverging results can be ascribed to the properties of the redundant case markers used. In particular, the grammatical marker *pazz* used in the earlier study was significantly more salient perceptually than the case markers used here (nominative *-i* and accusative *-o*). The greater salience of the case marker, in conjunction with the fact that it was the only novel lexical item in the sentences likely enhanced its detectability (MacWhinney, 2001), thus attracting learners’ attention and making it more readily learned (Rácz, 2013; Ellis, 2017). To test whether the magnitude and direction of the effect of redundancy rely on the level of salience of the redundant cue(s), Experiment 2 attempts to bridge the gap between the two studies by following a procedure identical to Experiment 1, but this time using a more salient case marker, *pazz*. If the presence of a salient redundant linguistic cue can indeed benefit grammar learning, then we should see better learning outcomes than those found in Experiment 1.

## 3 Experiment 2

### 3.1 Method

#### 3.1.1 Participants

Participants were a new set of 30 native English speakers (*M*_age_ = 28.83, SD_age_ = 8.08; female = 14) recruited online via Prolific using the same screening criteria as in Experiment 1. All participants gave informed consent electronically before beginning the experiment and were compensated for their participation after the study.

#### 3.1.2 Materials and procedure

The lexicon and grammatical structure of the artificial language were identical to those of Experiment 1, with the exception that, similar to Tal and Arnon (2022) objects were marked with a post-nominal nonce grammatical marker (i.e., *pazz*), as in **(2)**. The procedure for Experiment 2 was identical to that described for Experiment 1.

**(2)** *Varek velg-pazz alg*

jump over velg-ACC alg

‘The alg is jumping over the velg.’

### 3.2 Results

**Figure 4:**
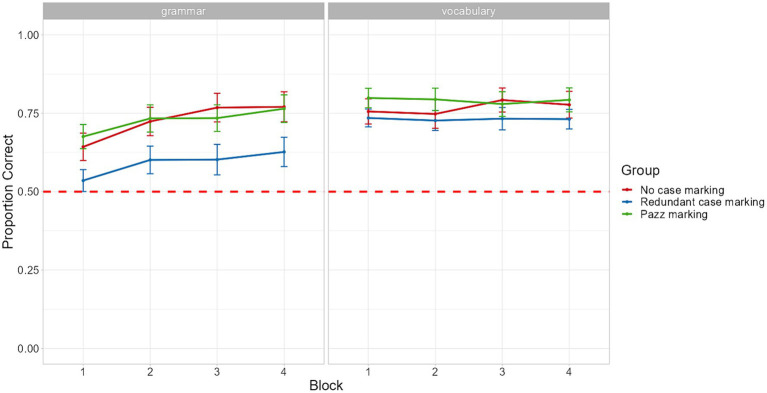
Performance across the four blocks of the sentence comprehension test for groups in Experiments 1 and 2. The red dashed line represents chance performance and error bars, calculated over by-subject means, represent standard errors of the mean.

Group scores for the training phases (Table 3) and the sentence comprehension test were calculated as in Experiment 1. A series of Kruskal-Wallis tests that were used to compare performance between this group and those of Experiment 1 in the three training phases indicated that there was a significant difference only in the first stage of the Sentence training phase (χ2(2) = 10.96 *p* = <0.01). Thus, again, no differences in the knowledge of the individual words, when presented in isolation, were detected among groups. This was followed by a pairwise Wilcoxon test (with Bonferroni correction) which showed that the *pazz* marking group of Experiment 2 had significantly higher scores than the no case marking group of Experiment 1 (W = 240, *p* < 0.01). The performance of each group during the sentence comprehension test is shown in Figure 4. As was the case for the two groups in Experiment 1, a strong correlation between vocabulary and grammar scores in the main comprehension test was also obtained for participants in the *pazz* marking group (*rho* = 0.79, *p* < 0.01).

**Table 3:** Mean performance (%) in the training phases in Experiment 2.

	Mean	SD	Median	IQR	Min-Max
Vocabulary pre-training stage 2	60.6	22.0	60.8	27.9	15–96.7
Sentence training stage 1	87.0	14.0	90.0	12.5	46.7–100
Sentence training stage 2	78.2	16.8	82.5	29.2	46.7–98.3
Sentence training stages 1 & 2	81.1	14.6	84.4	21.9	52.2–98.9

Participants’ responses on the trials testing grammar learning in the Sentence Comprehension test in both Experiment 1 and 2 were submitted to a second logistic mixed-effects regression model. The model had Response accuracy as the categorical dependent variable, Block (effect coded as block 1 = −1, block 2 = −0.5, block 3 = 0.5 and block 4 = 1), Vocabulary Learning, Group, coded using sliding difference contrasts (*Salience contrast*: redundant case marking versus *pazz* case marking, *Redundancy contrast*: *pazz* case marking versus no case marking), and all two- and three-way interactions between these variables as fixed effects. The model was initially fitted with the maximal random effects structure justified by the design but was eventually simplified by removing random slopes iteratively until a non-singular fit was obtained (Barr et al., 2013; Bates et al., 2015). Post-hoc (Bonferroni-corrected) analyses and measures of effect size (odds ratios and their 95% confidence intervals) were computed as in Experiment 1. The VIFs (GVIF^([1 = 2Df])) of all variables were below <2.00, indicating no multicollinearity issues. The results obtained from all post-hoc analyses are provided in Appendix C.

Table 4 presents the summary of the mixed effects model. A significant positive intercept was found indicating that, overall, participants across groups were able to accurately select the target referent, exhibiting learning of the novel VOS word order. In addition, there was a main effect of Block, suggesting that performance improved over time, and a main effect of Vocabulary Learning, such that participants who achieved higher levels of vocabulary knowledge, as indicated by their performance in the training phase, showed greater grammatical accuracy. There was also a two-way interaction between the Block and Vocabulary Learning, whereby post-hoc analysis suggested that the effect of Block was stronger for learners with higher vocabulary learning scores but was not significant for those who had low scores (−1SD).

**Table 4:** Results of the mixed-effects model for response accuracy in the grammar trials of the sentence comprehension test in Experiment 2.

Variable	$\hat{\beta }$	SE	*Z*	*p*	Odds ratios (CI)
(Intercept)	1.27	0.14	9.18	<0.001	3.56 (2.71–4.67)
Salience contrast – *pazz* marking vs. redundant case marking	0.49	0.32	1.51	0.132	1.63 (0.86–3.06)
Redundancy contrast – no case marking vs. *pazz* marking	0.42	0.33	1.29	0.197	1.52 (0.80–2.89)
Block	0.57	0.08	7.53	<0.001	1.77 (1.53–2.05)
Vocabulary learning	1.17	0.14	8.51	<0.001	3.23 (2.47–4.23)
Salience contrast – block	0.02	0.17	0.10	0.917	1.02 (0.73–1.42)
Redundancy contrast – block	0.46	0.18	2.50	0.012	1.58 (1.10–2.27)
Salience contrast – vocabulary learning	0.76	0.33	2.34	0.019	2.15 (1.13–4.08)
Redundancy contrast – vocabulary learning	0.26	0.33	0.78	0.433	1.29 (0.68–2.46)
Block: vocabulary learning	0.50	0.08	6.43	<0.001	1.64 (1.41–1.91)
Salience contrast – block: vocabulary learning	0.24	0.17	1.36	0.174	1.27 (0.90–1.79)
Redundancy contrast – block: vocabulary learning	0.19	0.19	1.03	0.304	1.21 (0.84–1.75)
*Random effects*	*Variance*	*SD*			
Item	0.07	0.26			
Participant	0.82	0.91			
Vocabulary learning | item	0.04	0.20			
Block | participant	0.16	0.40			

**Figure 5:**
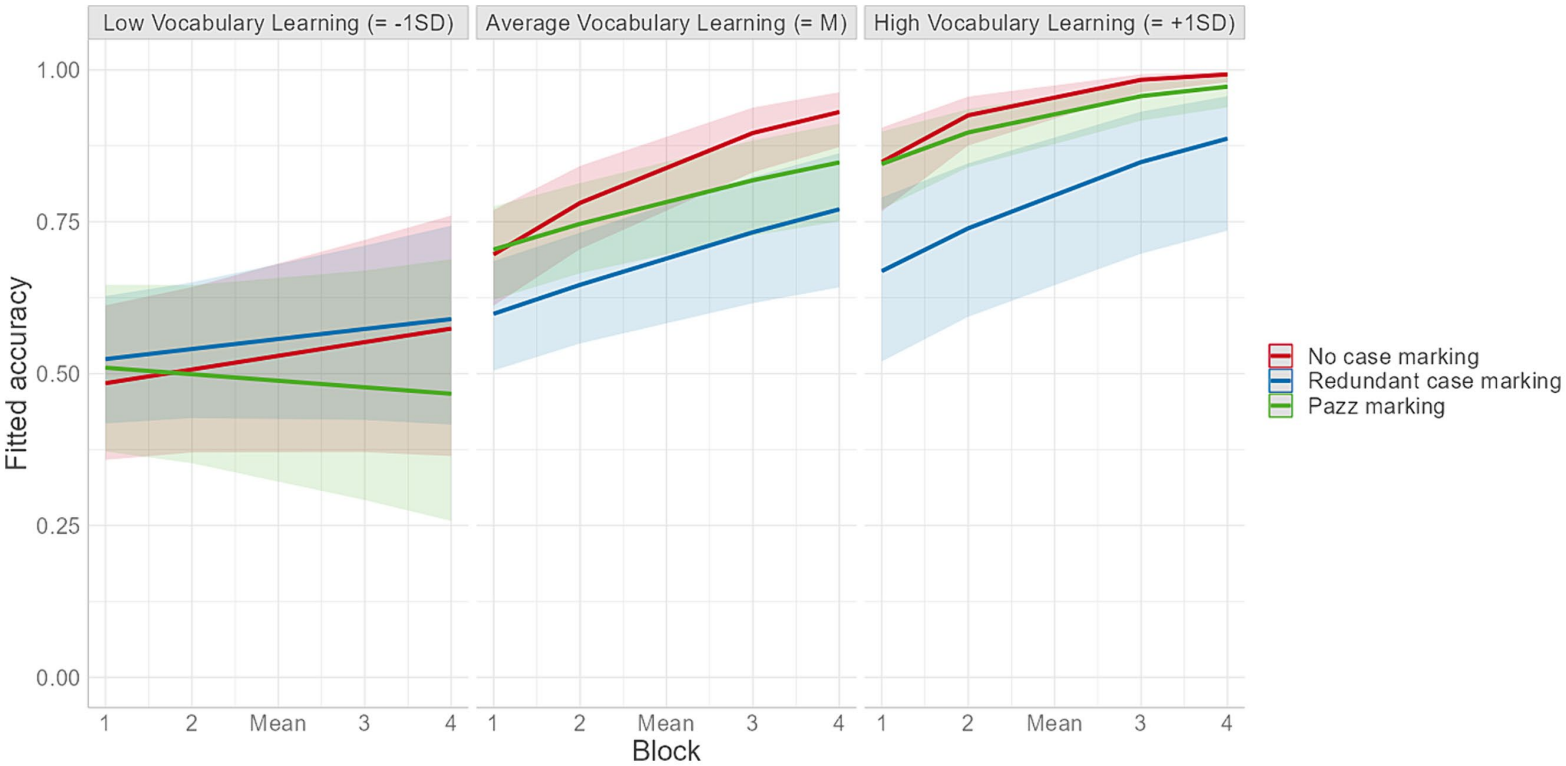
Fitted accuracy as a function of vocabulary learning and block for the three learning groups.

Turning to the sliding difference contrasts analyses, the model did not reveal any significant effect of Group and there was no interaction between redundant case marking versus *pazz* marking groups (salience contrast) and Block. Note that the contrast between the no case marking group and the low-salient redundant case marking group of Experiment 1 reached significance even in this larger model (see Appendix C). The interaction between *pazz* marking versus no case marking groups (redundancy contrast) and Block emerged as significant and was followed up by post-hoc analyses which showed that the effect of block was significantly stronger for the no case marking group (see Appendix C). In terms of the interactions of Vocabulary Learning with the two group contrasts, only that with the Salience contrast emerged as significant. Subsequent pairwise comparisons suggested that the differences in accuracy between the *pazz* marking and the redundant case marking conditions increased with increasing vocabulary learning scores in the sentence training phase, indicating that participants with high vocabulary scores (+1SD) in the *pazz* marking condition demonstrated significantly greater grammar learning gains compared to high vocabulary learners in the redundant case marking condition (Figure 5; see also Appendix C).

### 3.3 Discussion

In this experiment, a new group of learners were exposed to an artificial language where the redundant case marker *pazz*, as used in Tal and Arnon (2022), followed all objects. Similar to Experiment 1, the results of Experiment 2 showed that native speakers of English can quickly acquire a language with a novel word order (76.4% correct in block 4) and that participants’ grammar learning outcomes are strongly related to their vocabulary knowledge prior to the test phase, as learners with low vocabulary scores demonstrated negligible or no grammar gains. Most importantly, Experiment 2 set out to examine whether acquiring a redundant language is still more difficult to adults if the level of salience of the redundant case marker is increased. Results showed that learners in the *pazz* marking condition were quite accurate in comprehending the novel word order pattern, and, in fact, good vocabulary learners in this condition were significantly more accurate in comprehending the novel word order pattern than those who achieved high vocabulary learning rates in the low-salient redundant case marking condition of Experiment 1 (see Appendix C). However, the difference between the two conditions was found to dissipate with time and was no longer apparent towards the end of the test phase (block 4). On the other hand, no significant differences between the non-redundant group of the previous experiment and the pazz marking group were observed throughout the experiment. These findings indicate that the negative effect of the presence of redundant grammatical cues in the initial stages of exposure to a novel language observed in Experiment 1 appears to be less pronounced when the perceptual salience of these cues is increased. This appears to be in line with earlier research reporting that salience can be a key determinant of learners’ processing during early L2 learning (Ellis and Sagarra, 2010; Simoens et al., 2017).

## 4 General discussion

Using a fully artificial language paradigm, the experiments presented in this study sought to test the impact of the presence of redundant linguistic cues on L2 acquisition. In Experiment 1, two groups of adult native speakers of English were exposed to one of two artificial languages that exhibited the same word order, VOS, and shared an identical set of lexical items, but differed in that one of them also included redundant case markers to distinguish thematic roles. While both groups managed to learn the novel word order of the language, we found a hindering effect of redundancy on the comprehension of thematic role assignment, as participants in the non-redundant condition demonstrated greater grammar learning gains than those in the redundant condition. In Experiment 2, we manipulated the level of salience of the redundant linguistic cue by using a more perceptually salient marker, namely, *pazz,* the marker that was also used in Tal and Arnon (2022). Although, overall, the new redundant group and the low salient redundant group in the first experiment achieved similar learning outcomes, this pattern of performance did not apply uniformly to all learners. Specifically, a strong difference in favor of the former group was manifested when the comparison was limited to those participants who possessed better knowledge of the vocabulary of the novel language before the test phase commenced. Furthermore, and crucially, the performance of this new group did not differ from that of the no case marking group in Experiment 1 at any time point during the study.

Importantly, in both experiments, the differences in performance across groups were found only when vocabulary had been learned to a large extent. In the case of learners who failed or struggled to develop at least a relatively basic knowledge of the novel lexicon, grammar learning outcomes were minimal, if not absent, as can be seen in Figures 3, 5. This strong link between participants’ learning of the vocabulary items prior to the test and their development of knowledge about the grammatical regularities of the language appears to be a recurrent theme in artificial language studies (Martin and Ellis, 2012; Rebuschat et al., 2021; Kenanidis et al., 2023) and aligns well with previous findings from studies on L1 acquisition and ultimate attainment (Bates et al., 1988; Dąbrowska, 2018; Llompart and Dąbrowska, 2020). Irrespective of whether these two aspects of language are acquired simultaneously to the same extent or sequentially, efficient vocabulary learning appears to be necessary for getting the learning of basic syntactic information, such as how individual words can be combined to form sentences, off the ground, a claim that is at the core of usage-based accounts of language acquisition (e.g., Tomasello, 2003; Bybee, 2010).

It is worth reiterating here that the differences in performance on the grammar trials are highly unlikely to stem from differences in vocabulary learning across groups. As noted in the discussion of Experiment 1, all groups achieved comparable success rates both in the vocabulary pre-training task, where all lexical items were presented in isolation, and in the second stage of the sentence comprehension phase, which preceded the main comprehension test.

Overall, the results of this study demonstrate that adult learners are unlikely to benefit from the presence of redundant grammatical markers when learning novel linguistic structures. Conversely, they are more prone to experience difficulties with processing these cues. Such difficulties, however, are likely compounded by the relatively low salience of these markers, the conditions under which learning takes place, and the learners’ experience (or lack thereof) with cues that have a similar function in their L1. Recall that participants in this study were native speakers of English, a strict word-order language that does not mark case on nouns. The fact that grammatical markers are most often unstressed and short in length makes them hard to notice in the input, particularly when learners are not provided with instructions about the grammatical rules of the languages. Therefore, in principle, our findings are compatible with the idea that L2 learners prefer to process non-redundant cues over those that are less meaningful and redundant (VanPatten, 2006). Moreover, they also appear to be in line with the notion of learned attention (Ellis, 2006; Ellis and Sagarra, 2010), according to which, in naturalistic contexts, linguistic cues that are redundant, and thereby unnecessary to interpret, are less likely to be attended to by L2 learners and, thus, are often blocked by L1 biases and other more salient cues –in this case, word order.

Nevertheless, not all redundant markers appear to behave in the same way. The results of Experiment 2 suggest that the group of learners exposed to a language that had a highly salient redundant case marker (*pazz*) were not hindered by the marker (vs. the non-redundant group), in contrast to the redundant group of Experiment 1, who had to disambiguate between the less salient vowel suffixes *-i* and *-o*. This is in agreement with previous empirical findings on the effect of salience on L2 acquisition (Goldschneider and DeKeyser, 2001; Ellis and Sagarra, 2010; Simoens et al., 2017). Specifically, cues that are more salient tend to attract more attention, which places them in a more prominent position for entering subsequent processing. Thus, following our previous argument, it is likely that the higher salience of the redundant linguistic cues can increase their noticeability and lead to their quicker uptake and integration in L2 learners’ processing of sentences.

These results seem to extend and qualify the previous findings by Tal and Arnon (2022), who showed that redundancy can facilitate learning of thematic assignment in children and, crucially, failed to find a detrimental effect for adult learners. Recall that prior to the beginning of the present study, participants had no knowledge of the lexical items and the grammatical structure of the artificial language. Although, in this language, both word order and case marking were equally reliable, learners were more likely to rely on the former of the two cues, possibly due to L1 transfer effects from English. In the absence of other cues, participants in the non-redundant group continued to rely on word order to interpret the sentences throughout the task, thus achieving high levels of learning. However, participants in both redundant groups also had to process the redundant case marking cue. Given the lack of corrective feedback and information about the language’ structure, the mere presence of this redundant cue may have interfered with learners’ processing, causing (some of) them to shift away from the reliable word order cue in their effort to integrate the case marker, which likely required longer time for acquisition. Such a process was particularly taxing for the redundant group of Experiment 1, since the lack of salience of the redundant cue, at least relative to that used in Experiment 2, rendered it difficult for them to perceive the marker from the input and analyze it. Moreover, participants in this condition were presented with sentences containing two case markers, the previously learned (pre-trained) nominative case marker *-i*, which was the default form, and the accusative marker *-o*. Attending to the distinction between the two and identifying its functionality in the sentence might have exacerbated the negative redundant cue effect. This indicates that when a language has to be learned all from scratch, and under incidental conditions, redundancy is unlikely to be beneficial for adult learners, at least during the very first stages of learning.

There are two further things that warrant attention in relation to the present findings and those reported by Tal and Arnon (2022). First, in contrast to our participants, the learners in Tal and Arnon (2022) were already familiar with the individual words and their referents (as well as, to a certain extent, with the target grammatical structure, given that OSV can occur in Modern Hebrew) and, consequently, managed to quickly focus their attention on the redundant marker. In that study, it is therefore quite likely that, by the start of the test phase, participants in the redundant condition had already acquired the two cues to thematic role assignment (word order and case marking) and were able to draw from both or, conceivably, either of them to determine the agent/patient roles, as opposed to the non-redundant group for which only one cue was available. Indeed, the high accuracy rates after only 12 familiarization trials indeed suggest that, overall, learners had little trouble acquiring the relevant cues. Lastly, there is an argument to be made as to the extent to which such a learning situation may actually mirror natural language acquisition, for it is difficult to envisage a situation in which learners that already possess well-developed knowledge of the lexical items of a language and are also familiar with its grammatical structure encounter a reliable (and salient) grammatical cue for the first time. A second point, and potentially a corollary of the methodological discrepancies in the two studies, pertains to the differences in performance between learners in the *pazz* marking and the no case marking conditions observed in the present study. Though we did not find a difference across the board between the two groups, learners in the latter group were significantly more likely to demonstrate improved performance as the test progressed. This indicates that, irrespective of their salience, redundant markers may come with a processing cost at the initial stages of L2 learning.

Thus, by showing that redundancy fails to facilitate language acquisition among adult learners, the results of this study appear to be in accordance with the tenets of the Linguistic Niche Hypothesis (Lupyan and Dale, 2010; Dale and Lupyan, 2012). Although not tested here, the facilitative effect of redundant cues found in Tal and Arnon (2022) was significant for children, suggesting that this group may benefit from the presence of such cues, but their results were less conclusive for adults. Hence, our findings extend previous work and augment the argument for the differential impact of redundant cues on learners of different ages. Nevertheless, whether similar, positive redundancy effects would also be obtained when exposing child learners to a novel and more complex language, like the one used here, still remains to be seen.

The fact that no evidence for an advantage of redundant marking emerged in this study does not necessarily mean that such cues are not altogether helpful or not exploited by L2 learners. Instead, an alternative interpretation of these findings is that the effect of redundancy may depend on the stage of L2 acquisition that learners are at; the presence of redundant items may hinder grammar processing (and acquisition) at the very initial stages, like in this study, but it may serve to aid processing of novel or infrequent/non-canonical constructions at more advanced stages of the acquisition process, once learners have developed a basic knowledge of the new language, by enhancing the robustness of the utterance’s meaning (Aylett and Turk, 2004; Gibson et al., 2019; cf. Appendix A). However, it must be acknowledged that the type of (perfect) redundancy tested in these studies is extremely rare in natural languages, where linguistic cues are typically probabilistic (Chater and Manning, 2006; Monaghan, 2017). Nevertheless, the reported results provide important insights into the functional role of redundancy in L2 acquisition and the learners’ ability to process these redundant cues at different stages of the acquisition process.

In sum, our results suggest that, at the very early stages of L2 acquisition, redundancy is likely to impede learning of novel grammatical structures in adults, but the hindering effect they may experience can be, to some extent, mitigated by the level of perceptual salience of the redundant linguistic cues. Still, despite of this positive impact of salience, form-focused instruction appears to remain important, if not necessary, to facilitate the acquisition of forms and cues that are redundant. Indeed, a number of studies have demonstrated that various types of instruction, ranging from text enhancement (Cintrón-Valentín and Ellis, 2016) to explicit metalinguistic feedback or explanation (Ellis et al., 2006), can promote noticing of target L2 forms that are less salient (and redundant) by drawing learners’ attention to them and enhancing their processing.

Before concluding, it should be noted that, while a number of explanations were put forward here to account for the role of redundancy in early L2 acquisition, some of them remain speculative, calling for further research. For instance, while we have suggested that the impact of grammatical redundancy can be influenced by learners’ familiarity with the target grammatical structure from their earlier L1 experience and the nature of the artificial language employed, further investigation would be needed to confirm these hypotheses. This could potentially take the form of a replication study involving a semi-artificial language that contains the case markers used here (i.e., -*i* and -*o*), as suggested by a reviewer, or of an extension of the current study to include groups of learners from different L1 backgrounds. Future studies would also benefit from including larger sample sizes, allowing to increase statistical power, and a longitudinal design which could potentially pinpoint variations in the effect of redundancy across the L2 learning trajectory.

## Data availability statement

The datasets presented in this study can be found in online repositories. The names of the repository/repositories and accession number(s) can be found at: https://osf.io/v82j4/?view_only=14f5f4d603a84cecb1dbf183d6d73e8a.

## Ethics statement

Ethical approval was not required for the studies involving humans because the regulations of our institution and funding body do not require the submission of an ethics application for studies with human subjects that involve no more or less than only minimal risk. The studies were conducted in accordance with the local legislation and institutional requirements. The participants provided their written informed consent to participate in this study. Written informed consent was obtained from the individual(s) for the publication of any potentially identifiable images or data included in this article.

## Author contributions

PK: Conceptualization, Data curation, Formal analysis, Investigation, Methodology, Project administration, Visualization, Writing – original draft. ML: Conceptualization, Formal analysis, Methodology, Supervision, Writing – review & editing. SS: Conceptualization, Investigation, Methodology, Writing – review & editing. ED: Conceptualization, Funding acquisition, Methodology, Supervision, Writing – review & editing.

## Footnotes

^1^ https://app.gorilla.sc/openmaterials/571661

^2^It should, however, be noted that in modern Hebrew the accusative case is marked overtly by the free morpheme *et* which only precedes definite nouns (Berman, 1978).
