# Supplementary material for: Redundancy can hinder adult L2 grammar learning: evidence from case markers of varying salience levels
Source: Front Psychol. 2024 May 22;15:1368080. doi: 10.3389/fpsyg.2024.1368080 (PMC11150671; doi:10.3389/fpsyg.2024.1368080)
Supplement: Supplementary file 1 [file Table_1.DOCX]

Appendix A.

**Table 1.** Results on studies employing redundant linguistic cues in earlier studies.

**Study** **Participants** **Language** **Morphosyntactic target** **Assessment** **Result**

Jegerski (2015) Exp 1.

Exp. 2

Jegerski (2021) Exp 2.

1. near-native L1 English - L2 Spanish adults (mean age = 40.9)

16 near-native L1 English - L2 Spanish adults (mean age = 37.9)

20 intermediate level L1 English - L2 Spanish adults (mean age = 29.0)

Spanish Differential Object Marking Self-paced reading

Spanish Differential Object Marking Self-paced reading

Spanish Differential Object Marking Self-paced reading

No evidence of native-like sensitivity to violations of DOM when sentences did not contain the preverbal clitic *lo*

Native-like sensitivity to violations of DOM when sentences contained the

preverbal clitic *lo*

No evidence of online sensitivity to DOM even when it was doubled with the preverbal clitic *lo*.

| Taraban (2004) | Exp. 1 | 16 young adults | Artificial language | Locative phrases (noun + | Written production |  |
| --- | --- | --- | --- | --- | --- | --- |
|  |  |  |  |  |  |  |
|  |  |  |  | preposition) |  |  |

| Brooks et al. (1993) | Exp. 1 | 16 young adults | Artificial language | Locative phrases (noun + | Aural production |  |
| --- | --- | --- | --- | --- | --- | --- |
|  |  |  |  |  |  |  |
|  |  |  |  | preposition) |  |  |

| Exp.2 | 8 children aged 9;0 – 10;10 | Artificial language | Locative phrases (noun + | Aural production |  |
| --- | --- | --- | --- | --- | --- |
|  |  |  |  |  |  |
|  |  |  | preposition) |  |  |

Higher accuracy rates on items carrying a redundant phonological marker compared to unmarked items

Higher accuracy rates on items carrying a redundant phonological marker compared

to unmarked items

Higher accuracy rates on items

carrying a redundant phonological marker compared to unmarked items

**Table 1.** continued

**Study** **Participants** **Language** **Morphosyntactic target** **Assessment** **Result**

| Ellis & Sagarra (2010) | Study 3 L1 English - L2 Spanish adults | | Spanish | Cues for temporal reference | Reading eye-tracking |  |
| --- | --- | --- | --- | --- | --- | --- |
|  |  |  |  |  |  |  |
|  | • | low proficiency level |  | (adverb + verb inflection) | task |  |
|  | • | intermediate proficiency level |  |  |  |  |

- Higher levels of lexical cue use in non-native speakers.
- L2 proficiency effects (intermediate learners regressed to the adverb less than beginners)

| Sagarra & Ellis (2013) | L1 English - L2 Spanish adults | | Spanish | Cues for temporal reference | Reading eye-tracking |  |
| --- | --- | --- | --- | --- | --- | --- |
|  |  |  |  |  |  |  |
|  | • | 31 low proficiency |  | (adverb + verb inflection) | task |  |
|  | • | 30 high proficiency |  |  |  |  |

L1 Romanian - L2 Spanish

- 31 low proficiency
- 30 high proficiency
- L1 effects: Higher use of verbal morphology in Romanian than English learners to resolve tense incongruencies.
- L2 proficiency effects: L1 differences evident in high-, but not low-, proficiency L2 learners

Fedzechkina et al. (2017) 40 L1 English adults

Tal & Arnon (2022) 60 L1 Hebrew children

56 L1 Hebrew adults

Artificial language

Artificial language

Transitive construction (case Oral sentence

marking in flexible vs. fixed production

word order languages)

Transitive construction (with • Picture selection

or without redundant case • Oral sentence

marking) production

Case marking was maintained in the language with flexible order and was dropped in the fixed order language

- Children: Better learning in the redundant language condition.
- Adults: no significant differences between conditions.
