# Supplementary material for: Redundancy can hinder adult L2 grammar learning: evidence from case markers of varying salience levels
Source: Front Psychol. 2024 May 22;15:1368080. doi: 10.3389/fpsyg.2024.1368080 (PMC11150671; doi:10.3389/fpsyg.2024.1368080)
Supplement: Supplementary file 2 [file Table_2.DOCX]

**Appendix B**

**Table 1.** Group accuracy: pairwise contrasts for Βlock.

| Contrast | Block | Estimate | *SE* | *z.ratio* | *p* |
| --- | --- | --- | --- | --- | --- |
| No case marking – Redundant case marking | 1 | 0.34 | 0.26 | 1.32 | .186 |
|  | 2 | 0.54 | 0.29 | 1.89 | .060 |
|  | 3 | 0.94 | 0.41 | 2.28 | .022 |
|  | 4 | 1.14 | 0.49 | 2.32 | .020 |

**Table 2.** Group accuracy: pairwise contrasts for levels of Vocabulary Learning.

| Contrast | Vocabulary Learning | Estimate | *SE* | *z.ratio* | *p* |
| --- | --- | --- | --- | --- | --- |
| No case marking – Redundant case marking | Low: 54.6 (-1SD) | -0.36 | 0.47 | -0.77 | .442 |
|  | Average: 68.0 (M) | 0.74 | 0.34 | 2.18 | .030 |
|  | High:81.3 (+1SD) | 1.83 | 0.50 | 3.67 | <.001 |

**Table 3.** Simple slopes of Block for each level of Vocabulary Learning.

| Vocabulary Learning | Block.trend | *SE* | *z.ratio* | *p* |
| --- | --- | --- | --- | --- |
| Low Vocabulary Learning (-1SD): 54.6 | 0.11 | 0.12 | 0.88 | 1.0 |
| Average Vocabulary Learning (M): 68.0 | 0.65 | 0.10 | 6.40 | <.001 |
| High Vocabulary Learning (+1SD): 81.3 | 1.18 | 0.16 | 7.25 | <.001 |

**Table 4.** Group accuracy: pairwise contrasts for Block and levels of Vocabulary Learning.

| Contrast | Block | Estimate | *SE* | *z.ratio* | *p* |
| --- | --- | --- | --- | --- | --- |
| Low Vocabulary Learning (-1SD): 54.6 | | | | | |
| No case marking – Redundant case marking | 1 | -0.26 | 0.35 | -0.73 | .464 |
|  | 2 | -0.31 | 0.40 | -0.78 | .435 |
|  | 3 | -0.41 | 0.56 | -0.74 | .462 |
|  | 4 | -0.46 | 0.66 | -0.70 | .483 |
| Average Vocabulary Learning (M): 68.0 | | | | | |
| No case marking – Redundant case marking | 1 | 0.34 | 0.26 | 1.33 | .185 |
|  | 2 | 0.54 | 0.29 | 1.89 | .059 |
|  | 3 | 0.94 | 0.41 | 2.29 | .022 |
|  | 4 | 1.14 | 0.49 | 2.33 | .020 |
| High Vocabulary Learning (+1SD): 81.3 | | | | | |
| No case marking – Redundant case marking | 1 | 0.93 | 0.37 | 2.53 | .012 |
|  | 2 | 1.38 | 0.41 | 3.36 | <.001 |
|  | 3 | 2.28 | 0.61 | 3.73 | <.001 |
|  | 4 | 2.73 | 0.74 | 3.70 | <.001 |
