# Supplementary material for: Redundancy can hinder adult L2 grammar learning: evidence from case markers of varying salience levels
Source: Front Psychol. 2024 May 22;15:1368080. doi: 10.3389/fpsyg.2024.1368080 (PMC11150671; doi:10.3389/fpsyg.2024.1368080)
Supplement: Supplementary file 3 [file Table_3.DOCX]

**Appendix C.**

**Table 1.** Estimates of group pairwise comparisons.

| Contrasts | Estimate | *SE* | *z.ratio* | *p* |
| --- | --- | --- | --- | --- |
| Pazz marking -Redundant case marking | 0.49 | 0.32 | 1.51 | .396 |
| No case marking -Redundant case marking | 0.91 | 0.33 | 2.75 | .018 |
| No case marking -Pazz marking | 0.42 | 0.33 | 1.29 | .590 |

**Table 2.** Estimates of slopes of block for each level of the group variable and pairwise comparisons.

| Group | Block Estimate | *SE* | *z.ratio* | *p* |
| --- | --- | --- | --- | --- |
| Redundant case marking | 0.41 | 0.12 | 3.31 | <.001 |
| Pazz marking | 0.42 | 0.12 | 3.48 | <.001 |
| No case marking | 0.88 | 0.14 | 6.29 | <.001 |
|  |  |  |  |  |
| Contrasts | Estimate | *SE* | *z.ratio* | *p* |
| Pazz marking -Redundant case marking | 0.02 | 0.17 | 0.10 | 1.0 |
| No case marking -Redundant case marking | 0.48 | 0.18 | 2.59 | .029 |
| No case marking -Pazz marking | 0.46 | 0.18 | 2.50 | .037 |

**Table 3.** Group accuracy: pairwise contrasts for levels of Vocabulary Learning.

| Contrast | Vocabulary Learning | Estimate | *SE* | *z.ratio* | *p* |
| --- | --- | --- | --- | --- | --- |
| Pazz marking - Redundant case marking | Low: 56.3 (-1SD) | -0.28 | 0.44 | -0.63 | 1.0 |
|  | Average: 69.7 (M) | 0.49 | 0.32 | 1.51 | .393 |
|  | High: 83.1 (+1SD) | 1.25 | 0.48 | 2.60 | .028 |
| No case marking - Redundant case marking | Low: 56.3 (-1SD) | -0.11 | 0.42 | -0.27 | 1.0 |
|  | Average: 69.7 (M) | 0.91 | 0.33 | 2.75 | .018 |
|  | High: 83.1 (+1SD) | 1.93 | 0.51 | 3.78 | <.001 |
| No case marking - Pazz marking | Low: 56.3 (-1SD) | 0.17 | 0.48 | 0.35 | 1.0 |
|  | Average: 69.7 (M) | 0.42 | 0.33 | 1.29 | .589 |
|  | High: 83.1 (+1SD) | 0.68 | 0.45 | 1.51 | .391 |

**Table 4.** Simple slopes of Block for each level of Vocabulary Learning.

| Vocabulary Learning | Block.trend | *SE* | *z.ratio* | *p* |
| --- | --- | --- | --- | --- |
| Low Vocabulary Learning (-1SD): 56.3 | 0.08 | 0.09 | 0.84 | 1.0 |
| Average Vocabulary Learning (M): 69.7 | 0.57 | 0.08 | 7.54 | <.001 |
| High Vocabulary Learning (+1SD): 83.1 | 1.07 | 0.12 | 8.66 | <.001 |

**Table 5.** Group accuracy: pairwise contrasts for Block and levels of Vocabulary Learning.

| Contrast | Block | Estimate | *SE* | *z.ratio* | *p* | | |  |
| --- | --- | --- | --- | --- | --- | --- | --- | --- |
| Low Vocabulary Learning (-1SD): 56.3 | | | | | |  |  |  |
| Pazz marking - Redundant case marking | 1 | -0.06 | 0.36 | -0.16 | 1.0 | | |  |
|  | 2 | -0.17 | 0.38 | -0.43 | 1.0 | | |  |
|  | 3 | -0.39 | 0.51 | -0.76 | 1.0 | | |  |
|  | 4 | -0.50 | 0.59 | -0.84 | 1.0 | | |  |
| No case marking - Redundant case marking | 1 | -0.16 | 0.34 | -0.47 | 1.0 | | |  |
|  | 2 | -0.14 | 0.37 | -0.37 | 1.0 | | |  |
|  | 3 | -0.09 | 0.49 | -0.18 | 1.0 | | |  |
|  | 4 | -0.06 | 0.56 | -0.11 | 1.0 | | |  |
| No case marking - Pazz marking | 1 | -0.10 | 0.39 | -0.26 | 1.0 | | |  |
|  | 2 | 0.03 | 0.42 | 0.08 | 1.0 | | |  |
|  | 3 | 0.30 | 0.55 | 0.54 | 1.0 | | |  |
|  | 4 | 0.43 | 0.64 | 0.67 | 1.0 | | |  |
| Average Vocabulary Learning (M): 69.7 | | | | | | | | |
| Pazz marking - Redundant case marking | 1 | 0.47 | 0.26 | 1.78 | .227 | | |  |
|  | 2 | 0.48 | 0.28 | 1.70 | .270 | | |  |
|  | 3 | 0.50 | 0.38 | 1.31 | .570 | | |  |
|  | 4 | 0.51 | 0.44 | 1.14 | .766 | | |  |
| No case marking - Redundant case marking | 1 | 0.43 | 0.27 | 1.62 | .317 | | |  |
|  | 2 | 0.67 | 0.29 | 2.35 | .057 | | |  |
|  | 3 | 1.15 | 0.39 | 2.93 | .010 | | |  |
|  | 4 | 1.39 | 0.46 | 2.99 | .008 | | |  |
| No case marking - Pazz marking | 1 | -0.04 | 0.26 | -0.15 | 1.0 | | |  |
|  | 2 | 0.19 | 0.28 | 0.68 | 1.0 | | |  |
|  | 3 | 0.65 | 0.39 | 1.68 | .279 | | |  |
|  | 4 | 0.88 | 0.46 | 1.92 | .165 | | |  |
| High Vocabulary Learning (+1SD): 83.1 | | | | | | | | |
| Pazz marking - Redundant case marking | 1 | 1.00 | 0.39 | 2.54 | .033 | | |  |
|  | 2 | 1.12 | 0.42 | 2.69 | .021 | | |  |
|  | 3 | 1.38 | 0.57 | 2.42 | .046 | | |  |
|  | 4 | 1.51 | 0.67 | 2.24 | .075 | | |  |
| No case marking - Redundant case marking | 1 | 1.02 | 0.41 | 2.51 | .037 | | |  |
|  | 2 | 1.47 | 0.44 | 3.38 | .002 | | |  |
|  | 3 | 2.38 | 0.61 | 3.89 | <.001 | | |  |
|  | 4 | 2.84 | 0.73 | 3.87 | <.001 | | |  |
| No case marking - Pazz marking | 1 | 0.03 | 0.35 | 0.07 | 1.0 | | |  |
|  | 2 | 0.35 | 0.38 | 0.93 | 1.0 | | |  |
|  | 3 | 1.00 | 0.55 | 1.84 | .200 | | |  |
|  | 4 | 1.33 | 0.66 | 2.01 | .134 | | |  |
